# Supplementary material for: Powerful decomposition of complex traits in a diploid model
Source: Nat Commun. 2016 Nov 2;7:13311. doi: 10.1038/ncomms13311 (PMC5097135; doi:10.1038/ncomms13311)
Supplement: Supplementary Information — Supplementary Figures 1-8, Supplementary Table 1, Supplementary note 1 and Supplementary References. [file ncomms13311-s1.pdf]

Supplementary Figure 1

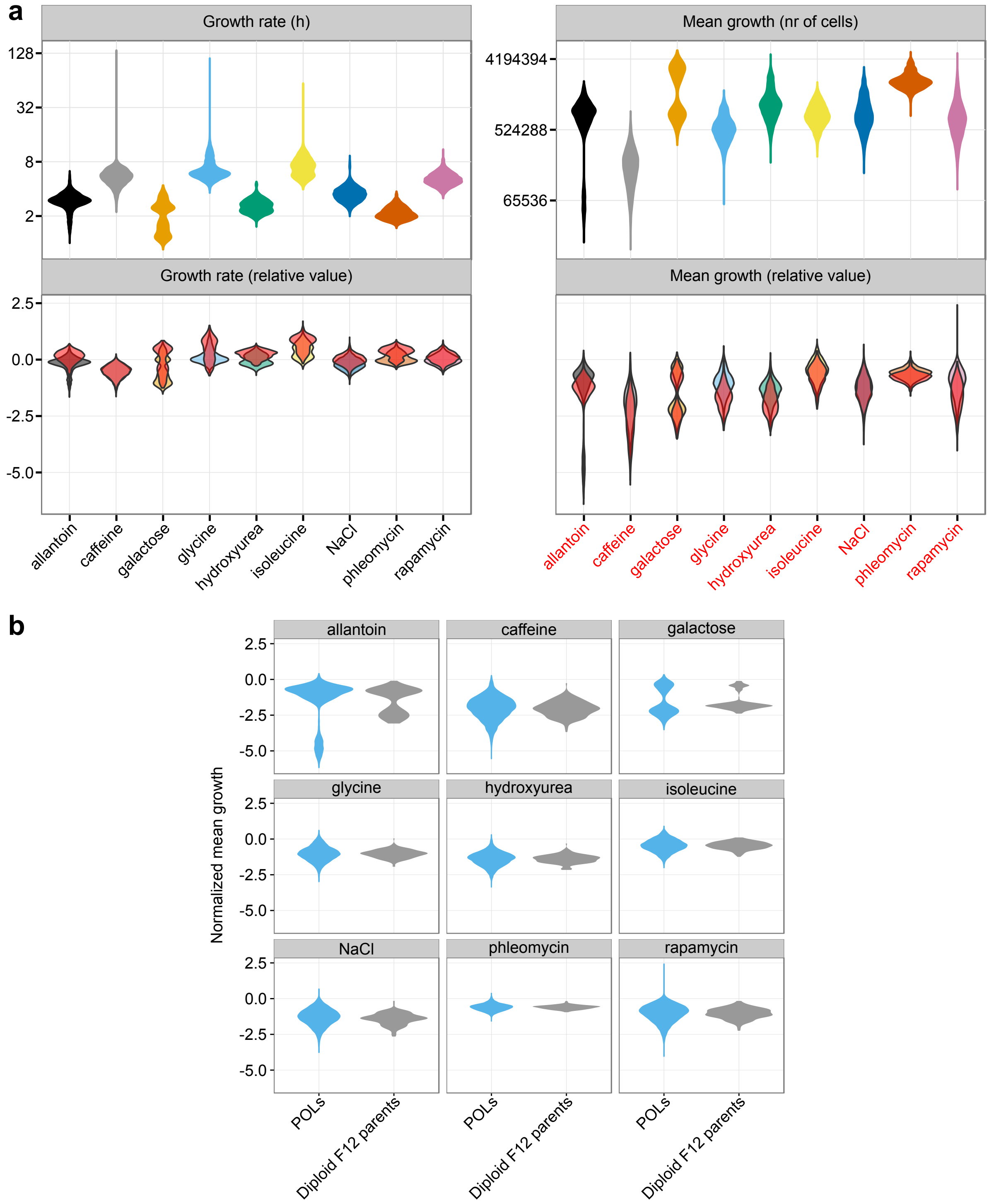

Supplementary Figure 1 | Growth phenotype distributions. (**a**) Variation in degree of stress in the different environments for maximum growth rate (left panel) and mean growth (right panel). In the upper panels actual population doubling time (hours), and mean growth (cells), are shown for all phenotyped strains to allow a direct biological interpretation of values. The transformation, from normalized trait values to actual doubling times and mean growth, was achieved by multiplying normalized values with the median control trait value and reversion of the log-transformation for each environment. In the lower panels, The same distributions have been divided to show the difference in distribution between strains without chr. IX aneuploidies (color coded by environment) and strains with chr. IX aneuploidies (red superimposed violins). (**b**) Frequency distribution of normalized phenotypes for POLs (blue) and estimated diploid F12 parents (gray).

Supplementary Figure 2

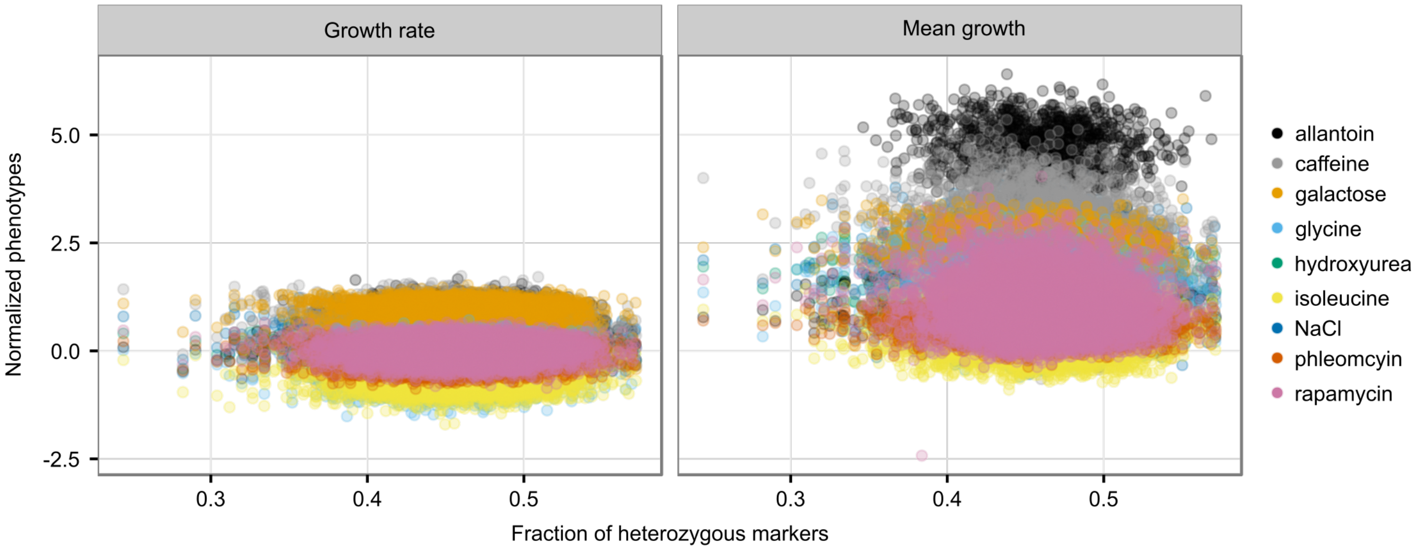

Supplementary Figure 2 | Extent of genome-wide homozygosity has no impact on yeast growth. Each point represents one POL in one environment (colors), with maximum growth rate (left panel) and mean growth (right panel) in different panels. x-axis shows mean heterozygosity, across all the segregating sites in the genome. y-axis shows the normalized growth phenotype.

Supplementary Figure 3

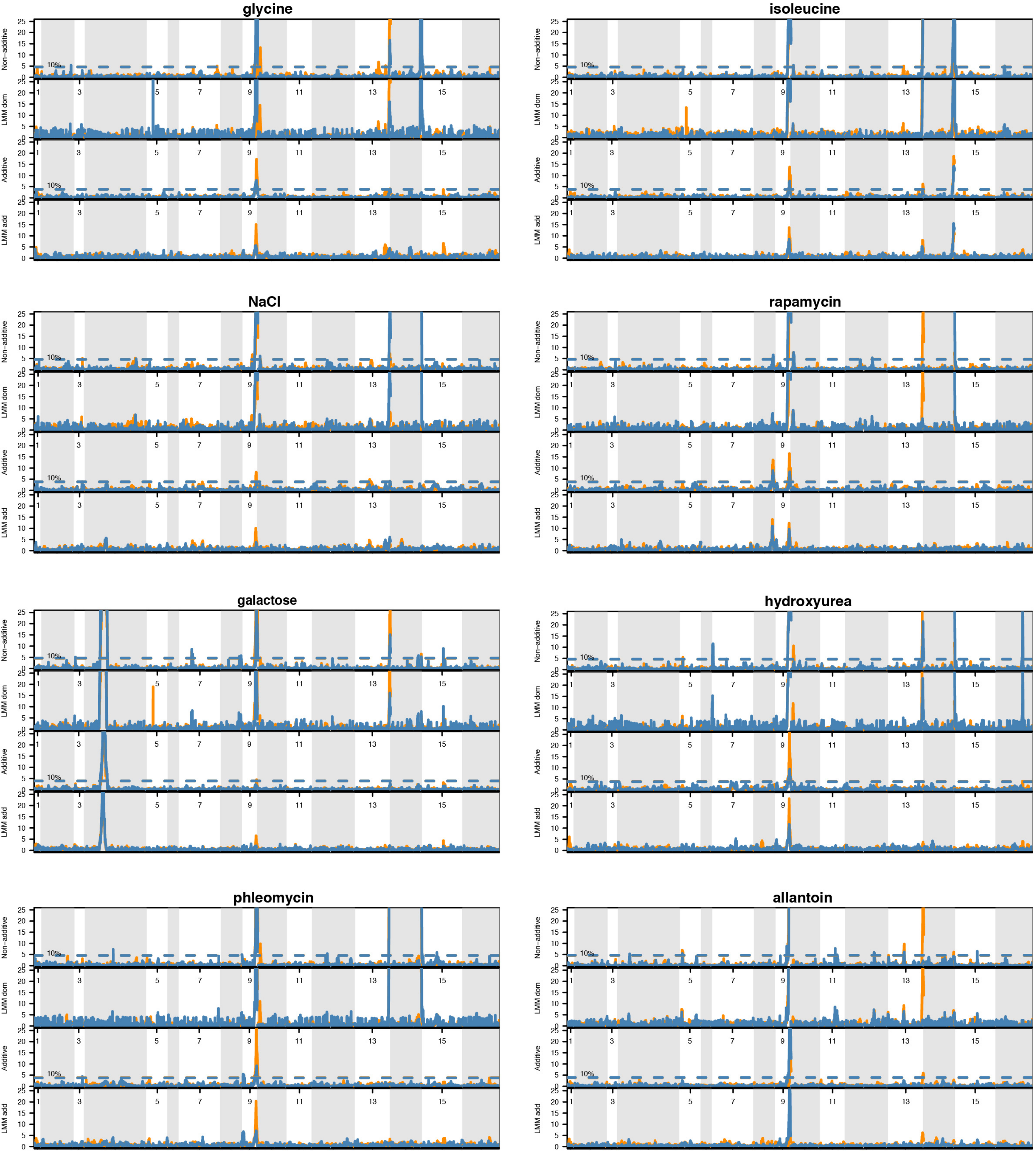

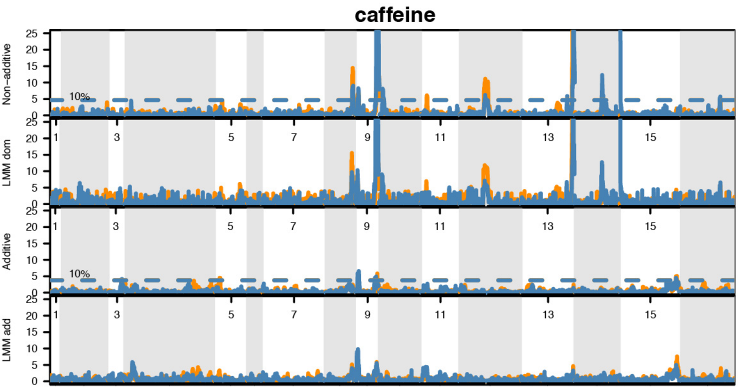

Supplementary Figure 3 | QTL maps for each trait. Four QTL mapping approaches were used for each trait: nonadditive QTL mapping, using residuals from the estimated diploid F12 parents (top panel); dominance, testing for significance of the dominance term using linear mixed models (second panel from the top); additive QTL mapping, using estimated diploid F12 parents (third panel from the top); and finally, additive QTL mapping, using linear mixed models (bottom panel). y-axis = LOD score, x-axis = genetic position. Orange: growth rate, blue: mean growth.

Supplementary Figure 4

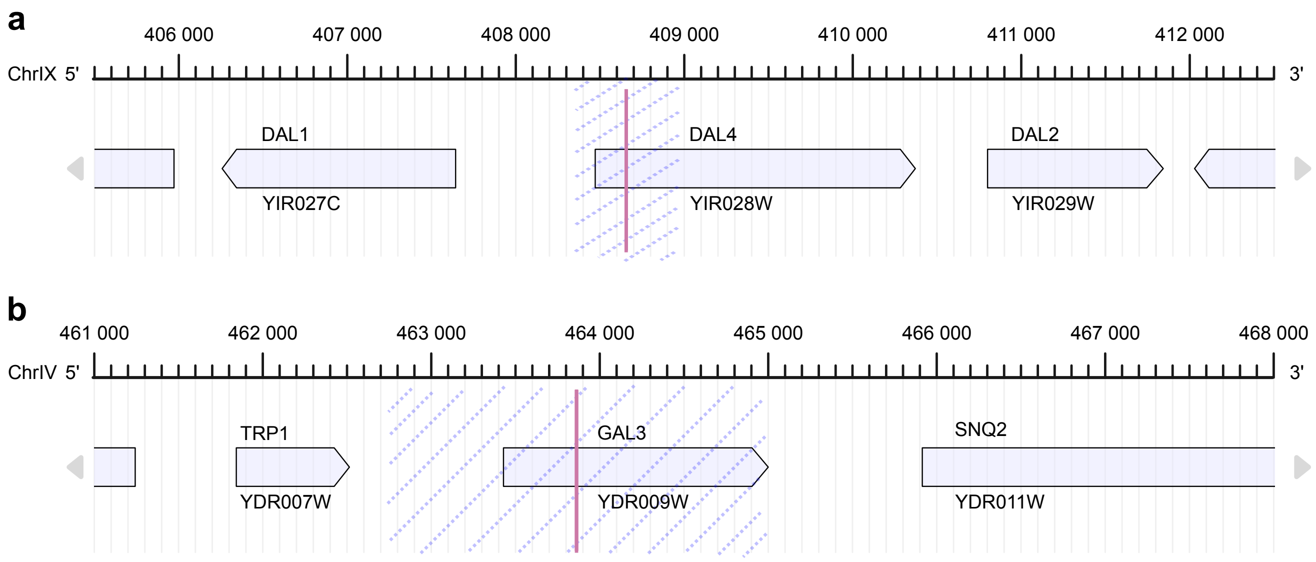

Supplementary Figure 4 | Major QTL positions. QTL positions (purple line) and associated 1.8-LOD support interval (dashed area) for (**a**) the chromosome IX QTL in allantoin and (**b**) the chromosome IV QTL in galactose. Both QTLs point to variants known to explain a large portion of the variation in the respective environments.

Figure initially constructed with AnnotationSketch (S. Steinbiss *et al.* AnnotationSketch, a library for drawing genome annotations. *Bioinformatics* **25(4),** 533-534 (2009)) and then modified.

Supplementary Figure 5

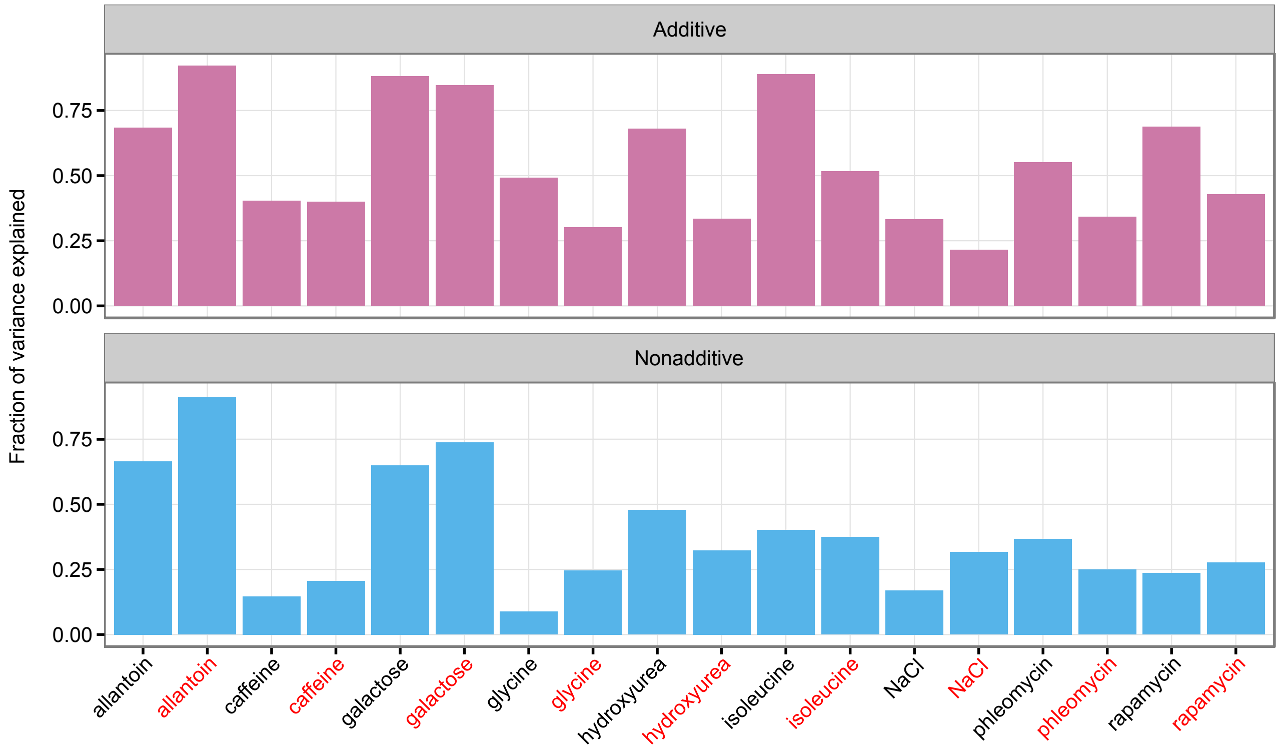

Supplementary Figure 5 | Variance explained by QTLs per environment. The accumulated variance explained by QTLs called in additive and nonadditive respectively. The variance explained by each QTL has been summed over each environment to show the fraction of variance explained by QTLs in each environment. Black labels = growth rate, red labels = mean growth.

Supplementary Figure 6

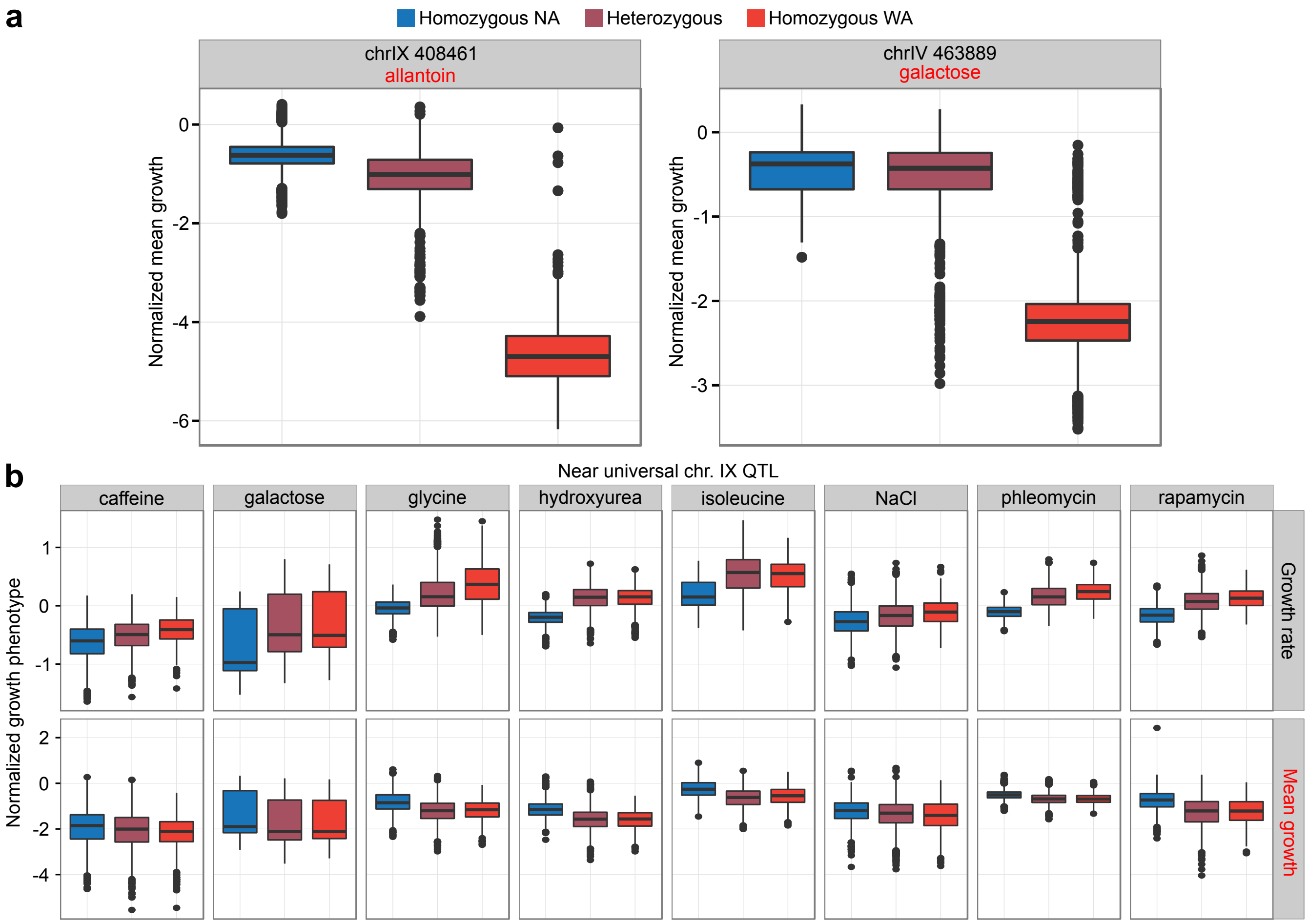

Supplementary Figure 6 | Phenotype distributions as a function of genotype for key QTLs. (**a**) Left panel: distribution of mean growth in allantoin, as a function of genotype composition at the QTL at chr. IX. Right panel: mean growth in galactose, as a function of genotype composition at the QTL on chr. IV. (**b**) Tukey boxplots for growth traits as a function of genotype composition at the near universal chr. IX QTL, penetrating in all but one evironment with antagonistic effects on maximum growth rate and mean growth. Note: on average, homozygote WA is the strongest genotype for maximum growth rate. However, as shown in Supplementary Figure 7, heterozygotes are heavily enriched among the best performing POLs.

Supplementary Figure 7

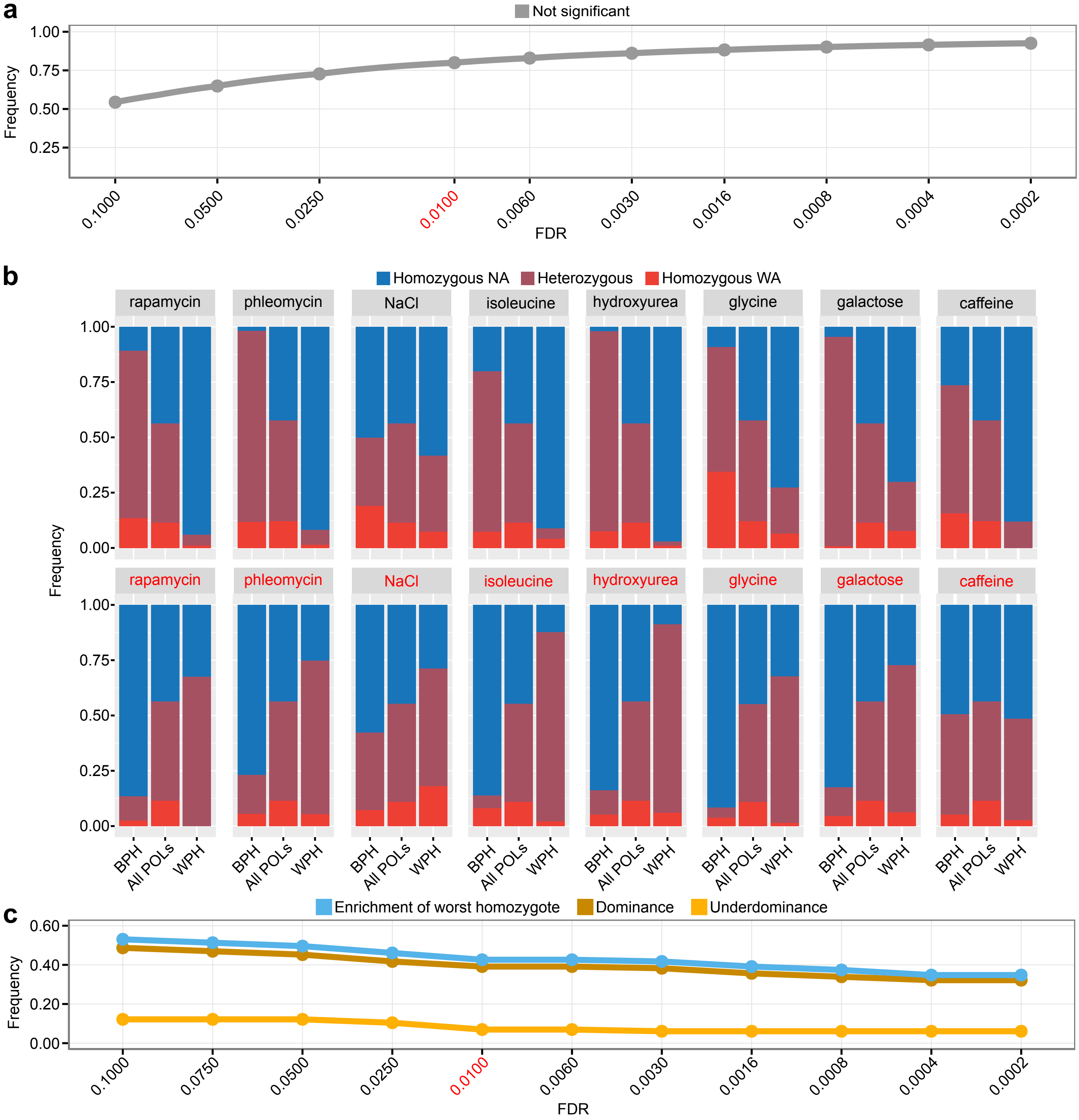

Supplementary Figure 7 | Dominance, overdominance, and underdominance in heterotic POLs. (**a**) Frequency of POLs not significantly different from their corresponding estimated diploid parents (y-axis) as a function of different FDR q-values (x-axis) where the red label (0.01) indicates the FDR q-value used for downstream analysis. (**b**) For each growth phenotype (black label = growth rate, red label = mean growth) the genotype frequencies for best parent heterotic POLs (BPH), all POLs and worst parent heterotic POLs (WPH) at the pleiotropic chr. IX QTL. Best parent heterotic POLs for growth rate at this segregating site are significantly overrepresented for the heterozygous genotype compared to all POLs in most environments ($p<0.01$, exception of NaCl, glycine and caffeine). Conversely, best parent heterotic POLs in mean growth are in most environments overrepresented for homozygous NA (χ^2 test, $p<0.01$, exception of caffeine) and underrepresented for the heterozygote (exception of NaCl and caffeine). (**c**) The percentage of WHP explained by enrichment of worst homozygote, dominance and underdominance as a function of FDR. Note: we show the outcomes of a range of FDR cut-off values to illustrate the robustness of conclusions; the cut-offs used for downstream analysis was set beforehand and not influenced by the results.

Supplementary Figure 8

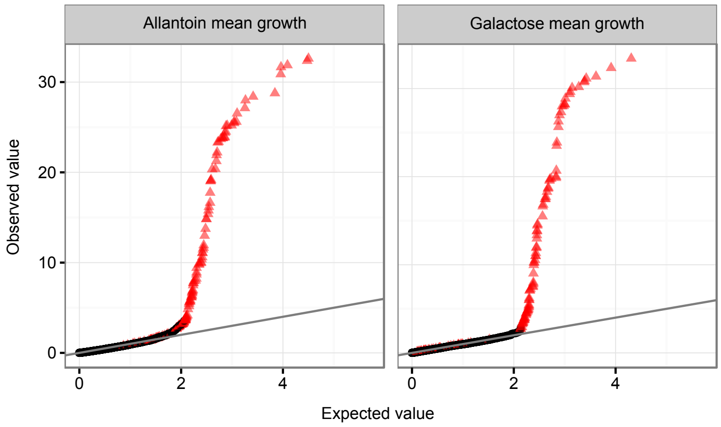

Supplementary Figure 8 | QQ-plots showing correction for population structure. This QQ-plot shows the expected vs. observed *p*-values for two of the QTL calls using linear mixed models. The locus with the strongest effect (red triangles) explain the outliers for the expected uniform distribution under the null.

Supplementary Table 1

Challenge	Conc.	Type	Cellular effect
NaCl	1.4M	Cation stress	Extracellular Na^+ exposes yeast to hyperosmosis, and requires intracellular glycerol accumulation and cell wall and cytoskeleton strengthening[1]. Na^+ enters cells through the K^+ transporters Trk1 and Trk2, and potentially through Pho89 and Nsc1. Intracellularly, it displaces K^+, challenging cell volume regulation, intracellular pH and membrane potentials, protein synthesis, and enzyme activation[2]. At acidic pH, Na^+ is exported by Nha1[3]. At higher pH, Na^+ efflux is mediated by the Ena proteins, encoded in a single gene in most natural strains but in Wine/European strains a different gene variant introgressed from *S. paradoxus* has been amplified into 3-5 similar paralogs[4]. This introgression/duplication largely defines natural yeast variation in salt tolerance. The Ena1 variant plays the critical role in Na^+ tolerance[5]. Regulation of salt tolerance genes is complex, involving the *HOG1*, Calcineurin pathway, *TOR* pathway, *RIM101* and glucose repression pathways.
Galactose	2%	Carbon source (replaces glucose)	Galactose well supports yeast growth as only energy and carbon source. It enters cells through the Gal2 permease in a process that also requires Gal1. Intracellular galactose is converted into glucose-1-phosphate by three sequential reactions that are catalyzed by Gal10, Gal1 and Gal7 respectively, with Gal10 also being required for re-cycling of a pathway intermediate. All the galactose structural genes are coordinately regulated at the level of transcription in response to galactose by Gal4p, Gal80p, and Gal3p[6,7]. All seven GAL genes are spatially co-localized in a gene cluster[8]. Large natural variations in galactose use is largely accounted for by loss-of-function mutations in Gal2, Gal3[4] or loss of the whole GAL cluster[8].
Caffeine	2.25mg/mL	Toxin	A purine, similar to adenine and guanine, that binds multiple enzymatic targets. In yeast, caffeine targets the TORC1 complex[9], caffeine prevents gene conversion by displacing Rad51[10] (Tsabar et al. 2015), impairs DNA repair, and may interfere with cell wall generation. Mutations in very diverse cellular components modulate caffeine sensitivity. Caffeine trafficking is not well understood.
Rapamycin	0.024µg/ml	Toxin	A polyketide macrolide produced by *Streptomyces hygroscopicus*. Rapamycin binds the Fpr1 protein[11]. This protein-drug complex binds to and inhibits Tor1[12], but not Tor2[13]. Tor1p is a component of TORC1, a protein complex regulating nutrient availability and stress responses[14]. Mutations in Fpr1 or Tor1 confer resistance to the drug[12]. Loss of non-essential TORC1 components (e.g. Kog1 and Tco089), or in TORC1 interactors (e.g. Rrd1) confer rapamycin sensitivity. Rapamycin binds to the Fpr1 paralog Fpr2 without known toxic effects[15]. Rapamycin trafficking is not well understood.
Phleomycin	2µg/ml	Toxin	A 12 component drug complex isolated from *Streptomyces*. Binds to DNA, impedes DNA polymerase and induces DNA damage and breakdown, potentially via an oxidative mechanism[16]. Arrests cells before entry into S-phase[17,18]. Loss of DNA repair components, such as Rad6, 9 or 17 cause phleomycin hypersensitivity[19,20]. Phleomycin trafficking is not well understood.
Hydroxyurea	2.5mg/ml	Toxin	Hydroxyurea inhibits reduction of ribonucleotides to deoxidized ribonucleotides[21], by binding to and inhibiting the four component RNR (ribonucleotide reductase) complex. dNTP depletion impedes synthesis and repair of DNA[22,23], arresting cells in early S-phase. The RNR complex consists of four proteins, RNR1, 2, 3, and 4, of which RNR1 and 2 are essential, and RNR4's essentiality depends on the genetic background[24-26]. Natural variation in hydroxyurea resistance is partially mediated via the Hur1 protein[4].
Glycine	30mg N/L	Nitrogen	Glycine is generally a very poor nitrogen source for yeast, but the growth

		source (replaces ammonium)	delays, rates and efficiencies vary between strains[27]. Glycine has no dedicated high affinity permease but is taken up by the broad specificity amino acid permeases Gap1[28] and Agp1 and the more specialized Dip5 and Put4[29]. Intracellular glycine is catabolized into ammonium in three sequential mitochondrial reactions. These are catalyzed by a single glycine cleavage complex with four components: Gcv3, Gcv1, Gcv2 and Lpd1. Cleavage reactions requires a folic acid derivative.
Isoleucine	30mg N/L	Nitrogen source (replaces ammonium)	Isoleucine is generally a poor nitrogen source for yeast, but growth delays, rates and efficiencies vary between strains[27]. Isoleucine is take up by the paralogous high affinity permeases Bap2[30] and Bap3[29] as well the low affinity general amino acid permease Gap1[28]. Isoleucine is catabolized in a single step reaction to glutamate, using the mitochondrial Bat1 or the cytoplasmic Bat2[31], with Bat1 expressed during exponential growth and Bat2 in stationary phase.
Allantoin	30mg N/L	Nitrogen source (replaces ammonium)	Allantoin is the primary nitrogen secretion product of higher mammals, excluding apes. Yeast utilizes allantoin as sole nitrogen source, but with varying delays, rates and efficiencies that largely maps to variation in Dal4 and Dal1[27]. The Dal4 permease is the only known entrance mechanism for allantoin. Intracellular allantoin is catabolized to urea in three sequential reactions catalyzed by Dal1, Dal2 and Dal3 respectively[32-34]. Urea is converted into ammonium by the multi-step enzyme Dur1,2[35]. The allantoin catabolic genes are regulated by both general and specific signals that involve Gln3, Gat1, Dal80, Dal81, and Dal82[36]. The *DAL* genes are encoded in a tight gene cluster on chr. IX[37].

Supplementary Note 1

1 Variance Decomposition Model

The precision with which one can estimate variance components from interactions increases in populations descending from a small number of founders[38]. We exploit the information about non-additive variation that is contained in a large cross between two different yeast strains to decompose the phenotypic variance of growth traits into components that come from additive effects, dominance effects, and pairwise and third order interaction effects.

We first introduce the random effects model, which gives the phenotypic covariance matrix as a linear combination of covariance matrices which reflect the covariance due to additive, dominance, pairwise, and third order interaction effects. The coefficients of this linear combination reflect the decomposition of the phenotypic variance into components originating from different types of genetic effect.

Let Y be a normalised phenotype, and let X be an observed and normalised $[N \times n_g]$ genotype matrix. Under a standard additive random effects model (see Yang *et al.*, 2010)[39],

$$Y = X\beta + \epsilon;\ \beta \sim \mathcal{N}(0, h^2/n_g);\ \epsilon \sim \mathcal{N}(0, \sigma^2). \tag{1}$$

Assuming that X is observed, and that β and ϵ are independent, this implies that

$$\mathrm{Cov}(Y) = h^2 \frac{1}{n_g} XX^T + \sigma^2 I. \tag{2}$$

Let

$$R = \frac{1}{n_g} XX^T = \frac{1}{n_g} K,\ \text{with}\ R_{i,j} = \frac{1}{n_g} \sum_{k=1} x_{ik} x_{jk}, \tag{3}$$

where x_{ik} is the normalized genotype of individual i at locus k. R is commonly termed the relatedness matrix, and gives the covariance between individuals due to additive effects of measured genotypes when scaled by h^2. $K = XX^T$ is the relatedness matrix un-normalised by the number of loci.

If one assumes that environmental similarity is uncorrelated with genetic similarity, and that all genotypes have been observed, then estimates of h^2 correspond to estimates of the narrow sense heritability of the phenotype Y.

Consider now a more general phenotype model which includes pairwise interactions and dominance effects.

$$Y_i = \beta_1 x_{i1} + \beta_2 x_{i2} + \delta_1 x_{i1}^m x_{i1}^p + \delta_2 x_{i2}^m x_{i2}^p + \beta_{12} x_{i1} x_{i2}; \tag{4}$$

where x_{i1}^m is the normalized maternally inherited allele of individual i at locus 1, and x_{i2}^p is the normalized paternally inherited allele of individual i at locus 2; δ_1 is the dominance effect of locus 1, and β_{12} is the interaction effect of loci 1 and 2; and

$$[\beta_1, \beta_2, \delta_1, \delta_2, \beta_{1,2}]^T \sim N(0, \mathrm{diag}(h^2/2, h^2/2, d^2/2, d^2/2, h_2^2)), \tag{5}$$

where d^2 is the proportion of variance due to dominance effects, and h_2^2 is the proportion of variance due to pairwise interactions. This assumes that the additive, dominance and interaction effects of the loci are uncorrelated. Therefore,

$$\begin{aligned}\mathrm{Cov}(Y_i, Y_j) =& h^2 \frac{1}{2}(x_{i1}x_{j1} + x_{i2}x_{j2}) + d^2 \frac{1}{2}(x_{i1}^m x_{i1}^p x_{j1}^m x_{j1}^p + x_{i2}^m x_{i2}^p x_{j2}^m x_{j2}^p) + \quad (6)\\ & h_2^2 x_{i1}x_{i2}x_{j1}x_{j2} + \mathrm{Cov}(\epsilon_i, \epsilon_j). \quad (7)\end{aligned}$$

We can recognise here element i, j of the additive relatedness matrix:

$$R_{i,j} = \frac{1}{2}(x_{i1}x_{j1} + x_{i2}x_{j2}). \quad (8)$$

Similarly,

$$\Delta_{i,j} = \frac{1}{2}(x_{i1}^m x_{i1}^p x_{j1}^m x_{j1}^p + x_{i2}^m x_{i2}^p x_{j2}^m x_{j2}^p) \quad (9)$$

is the element i, j of the dominance relatedness matrix, which in general for n_g loci is

$$\Delta_{i,j} = \frac{1}{n_g} \sum_{k=1}^{n_g} x_{ik}^m x_{ik}^p x_{jk}^m x_{jk}^p, \quad (10)$$

and can be calculated from diploid genomes without knowledge of parent of origin. Finally,

$$R_{i,j}^2 = x_{i1}x_{i2}x_{j1}x_{j2} \quad (11)$$

is element i, j of the matrix R^2 that, when scaled by h_2^2, gives the covariance due to pairwise interactions. In general, for n_g loci,

$$R_{i,j}^2 = \frac{2}{n_g(n_g-1)} \sum_{k=1}^{n_g} \sum_{l=k+1}^{n_g} (x_{ik}x_{il})(x_{jk}x_{jl}) = \frac{2}{n_g(n_g-1)} K_{i,j}^2, \quad (12)$$

where K^2 is R^2 un-normalized by the number of pairs of loci. This can be efficiently calculated given R by the formula

$$R^2 = \frac{1}{n_g(n_g-1)}[K \circ K - (X \circ X)(X \circ X)^T], \quad (13)$$

where $\circ$ represents the Hadamard product. The formula can be verified by calculation of element i, j.

This can be further generalised to third order interactions. The covariance due to third order interactions depends on the correlation between individuals across all triples of loci:

$$R_{i,j}^3 = \frac{6}{n_g(n_g-1)(n_g-2)} \sum_{k=1}^{n_g} \sum_{l=k+1}^{n_g} \sum_{m=l+1}^{n_g} (x_{ik}x_{il}x_{im})(x_{jk}x_{jl}x_{jm}). \quad (14)$$

This can be efficiently computed given R and R^2 by the formula:

$$R^3 = \frac{2}{n_g(n_g-1)(n_g-2)}[K^2 \circ K - K \circ (X \circ X)(X \circ X)^T + (X \circ X \circ X)(X \circ X \circ X)^T], \quad (15)$$

which can be verified by computing element i, j.

We therefore obtain an expression for the covariance matrix under a model allowing additive, dominance, pairwise interaction, and third order interaction random effects:

$$\text{Cov}(Y) = h^2 R + d^2 \Delta + h_2^2 R^2 + h_3^2 R^3 + \sigma^2 I, \quad (16)$$

where h_3^2 is the proportion of variance due to third order interactions. Given all the genotypes have been observed, R, Δ, R^2, R^3 can be computed in $O(nl^2)$ operations, and h^2, h_2^2, h_3^2, d^2, σ^2 can be fitted by restricted maximum likelihood in $O(n^3)$ operations.

2 Analysis of Yeast Cross

In the yeast cross, the environment is randomised, so genetic similarity should not be correlated with environmental similarity. Nearly all genetic variants genome wide have been observed by sequencing, so relatedness matrices calculated from genome wide genetic variation will capture nearly all of the genetic variance. The combination of these two properties makes the use of a linear mixed model with relatedness matrices calculated from observed genome wide genetic variants a legitimate way to estimate the variance decomposition of a phenotype.

2.1 Calculation of Covariance Matrices in Yeast Data

We compute the matrices R, Δ, R^2, R^3 for the yeast cross from genome wide genotypes determined by sequencing. We excluded genetic variants with greater than 1% missingness to prevent being overly influenced by noise.

Rare genetic variants can bias calculations of the relatedness matrix due to the normalisation procedure. We therefore excluded genetic variants with a frequency below 1%. This is unlikely to reduce heritability estimates by much as the variance explained by a variant is proportional to its variance.

For calculation of the dominance relatedness matrix, we first compute the normalized product of the maternal and paternal genotypes for each individual using the formula

$$x_{i1}^p x_{i1}^m = \frac{(g_{i1}^p - f_1)(g_{i1}^m - f_1)}{f_1(1-f_1)}, \quad (17)$$

where g_{i1}^p is the binary indicator variable for the minor allele on paternally inherited position 1 in individual i, and f_1 is the minor allele frequency at position 1. If f_1 is

known and the population has been randomly mating, the expectation of this is 0 and its variance is 1.

We estimated allele frequencies from the data and calculated $x_{i1}^p x_{i1}^m$ for all individuals and at all loci with minor allele frequency greater than 1% and missingness less than 1%. However, we further filtered loci which had sample expectations

$$\frac{1}{n}\sum_{i=1}^{n}\frac{(g_{i1}^p-\hat{f}_1)(g_{i1}^m-\hat{f}_1)}{\hat{f}_1(1-\hat{f}_1)} \tag{18}$$

which deviated by more than 0.05 from zero, the expectation under random mating and knowledge of allele frequencies. This mainly filtered out rare loci, owing to the sensitivity of the normalisation procedure to underestimation of low minor allele frequencies. It also may have filtered out loci with gross deviations from random mating, which could include loci with strong recessive effects which have been selected against. This may therefore cause an underestimation of dominance variance.

2.2 Simulations

We chose 100 loci evenly spaced across the genome with minor allele frequency greater than 5% and with no missing data. Each of these was given an additive effect. We then randomly chose ten of these to have dominance effects, and 50 pairs at random to have pairwise interaction effects. All effects were drawn from normal distributions.

We simulated 100 phenotypes with $h^2 = 0.5$, $d^2 = 0.1$ and $h_2^2 = 0.1$. We fitted the model

$$\mathrm{Cov}(Y) = h^2R + d^2\Delta + h_2^2R^2 + \sigma^2 I \tag{19}$$

in *GCTA*[40] using the Average Information algorithm. The results are in Table 1.

	Mean (SD) Estimates	Mean $\hat{SE}$
h^2	0.51 (0.026)	0.033
d^2	0.085 (0.008)	0.009
h_2^2	0.10 (0.015)	0.017

The columns are, from left to right, the sample mean (standard deviation) of the estimates, as well as the mean of the standard error estimates, from 100 simulated phenotypes. True values are $h^2 = 0.5$, $d^2 = 0.1$, $h_2^2 = 0.1$.

There is a slight underestimation of dominance variance likely to be due to removing loci which were not normalized properly.

2.2.1 With third order interactions

We then performed a simulation with the same number of loci with additive effects, dominance effects, and pairwise interactions, with an additional 50 triples of loci randomly chosen from the 100 causal loci to have third order interaction effects.

We simulated 200 phenotypes with $h^2 = 0.5$, $d^2 = 0.1$, $h_2^2 = 0.1$, and $h_3^2 = 0.1$. We fitted the model

$$\mathrm{Cov}(Y) = h^2 R + d^2 \Delta + h_2^2 R^2 + h_3^2 R^3 + \sigma^2 I \tag{20}$$

in *GCTA* using the Average Information algorithm. The results are in Table 2.

	Mean (SD) Estimates	Mean $\hat{SE}$
h^2	0.56 (0.037)	0.034
d^2	0.16 (0.023)	0.015
h_2^2	0.08 (0.022)	0.02
h_3^2	0.10 (0.021)	0.021

The columns are, from left to right, the sample mean (standard deviation) of the estimates, as well as the mean of the standard error estimates, from 100 simulated phenotypes. True values are $h^2 = 0.5$, $d^2 = 0.1$, $h_2^2 = 0.1$, $h_3^2 = 0.1$.

The inference for h_3^2 was accurate. However, the inference for the other variance components loses its accuracy when also fitting a third order component. It is not clear why this is, but it may be due to non-convexity of the likelihood function when fitting many highly correlated variance components. There is evidence for this in the bi-modality of the distribution of estimates for d^2 and h_2^2.

Supplementary References

1. Hohmann, S. Osmotic stress signaling and osmoadaptation in yeasts. *Microbiol. Mol. Biol. Rev.* **66,** 300–372 (2002).
2. Ariño, J., Ramos, J. & Sychrová, H. Alkali metal cation transport and homeostasis in yeasts. *Microbiol. Mol. Biol. Rev.* **74,** 95–120 (2010).
3. Bañuelos, M. A., Sychrová, H., Bleykasten-Grosshans, C., Souciet, J. L. & Potier, S. The Nha1 antiporter of Saccharomyces cerevisiae mediates sodium and potassium efflux. *Microbiology (Reading, Engl.)* **144 (Pt 10),** 2749–2758 (1998).
4. Warringer, J. *et al.* Trait variation in yeast is defined by population history. *PLoS Genet* **7,** e1002111 (2011).
5. Haro, R., Garciadeblas, B. & Rodríguez-Navarro, A. A novel P-type ATPase from yeast involved in sodium transport. *FEBS Lett.* **291,** 189–191 (1991).
6. Johnston, M. & Davis, R. W. Sequences that regulate the divergent GAL1-GAL10 promoter in Saccharomyces cerevisiae. *Mol. Cell. Biol.* **4,** 1440–1448 (1984).
7. Lohr, D., Venkov, P. & Zlatanova, J. Transcriptional regulation in the yeast GAL gene family: a complex genetic network. *FASEB J.* **9,** 777–787 (1995).
8. Slot, J. C. & Rokas, A. Multiple GAL pathway gene clusters evolved independently and by different mechanisms in fungi. *Proc. Natl. Acad. Sci. U.S.A.* **107,** 10136–10141 (2010).
9. Reinke, A., Chen, J. C.-Y., Aronova, S. & Powers, T. Caffeine targets TOR complex I and provides evidence for a regulatory link between the FRB and kinase domains of Tor1p. *J. Biol. Chem.* **281,** 31616–31626 (2006).
10. Tsabar, M., Mason, J. M., Chan, Y.-L., Bishop, D. K. & Haber, J. E. Caffeine inhibits gene conversion by displacing Rad51 from ssDNA. *Nucleic Acids Res.* **43,** 6902–6918 (2015).
11. Koltin, Y. *et al.* Rapamycin sensitivity in Saccharomyces cerevisiae is mediated by a peptidyl-prolyl cis-trans isomerase related to human FK506-binding protein. *Mol. Cell. Biol.* **11,** 1718–1723 (1991).
12. Heitman, J., Movva, N. R. & Hall, M. N. Targets for cell cycle arrest by the immunosuppressant rapamycin in yeast. *Science* **253,** 905–909 (1991).
13. Loewith, R. *et al.* Two TOR complexes, only one of which is rapamycin sensitive, have distinct roles in cell growth control. *Mol. Cell* **10,** 457–468 (2002).
14. Loewith, R. & Hall, M. N. Target of rapamycin (TOR) in nutrient signaling and growth control. *Genetics* **189,** 1177–1201 (2011).
15. Nielsen, J. B. *et al.* Yeast FKBP-13 is a membrane-associated FK506-binding protein encoded by the nonessential gene FKB2. *Proc Natl Acad Sci USA* **89,** 7471–7475 (1992).
16. Moore, C. W. Internucleosomal cleavage and chromosomal degradation by bleomycin and phleomycin in yeast. *Cancer Res.* **48,** 6837–6843 (1988).
17. Nakada, D., Shimomura, T., Matsumoto, K. & Sugimoto, K. The ATM-related Tel1 protein of Saccharomyces cerevisiae controls a checkpoint response following phleomycin treatment. *Nucleic Acids Res.* **31,** 1715–1724 (2003).
18. Weinert, T. A., Kiser, G. L. & Hartwell, L. H. Mitotic checkpoint genes in budding yeast and the dependence of mitosis on DNA replication and repair. *Genes Dev.* **8,** 652–665 (1994).
19. Belenguer, P., Oustrin, M. L., Tiraby, G. & Ducommun, B. Effects of phleomycin-induced DNA damage on the fission yeast Schizosaccharomyces pombe cell cycle. *Yeast* **11,** 225–231 (1995).
20. He, C. H., Masson, J. Y. & Ramotar, D. A Saccharomyces cerevisiae phleomycin-sensitive mutant, ph140, is defective in the RAD6 DNA repair gene. *Can. J. Microbiol.* **42,** 1263–1266 (1996).
21. Krakoff, I. H., Brown, N. C. & Reichard, P. Inhibition of ribonucleoside diphosphate reductase by hydroxyurea. *Cancer Res.* **28,** 1559–1565 (1968).
22. Yao, R. *et al.* Subcellular localization of yeast ribonucleotide reductase regulated by the DNA replication and damage checkpoint pathways. *Proc Natl Acad Sci USA* **100,** 6628–6633 (2003).
23. Koç, A., Wheeler, L. J., Mathews, C. K. & Merrill, G. F. Hydroxyurea arrests DNA replication by a mechanism that preserves basal dNTP pools. *J. Biol. Chem.* **279,** 223–230 (2004).
24. Elledge, S. J. & Davis, R. W. Identification and isolation of the gene encoding the small subunit of ribonucleotide reductase from Saccharomyces cerevisiae: DNA damage-inducible gene required for mitotic viability. *Mol. Cell. Biol.* **7,** 2783–2793 (1987).
25. Elledge, S. J. & Davis, R. W. Two genes differentially regulated in the cell cycle and by DNA-damaging agents encode alternative regulatory subunits of ribonucleotide reductase. *Genes Dev.* **4,** 740–751 (1990).
26. Huang, M. & Elledge, S. J. Identification of RNR4, encoding a second essential small subunit of ribonucleotide reductase in Saccharomyces cerevisiae. *Mol. Cell. Biol.* **17,** 6105–6113 (1997).
27. Ibstedt, S. *et al.* Concerted evolution of life stage performances signals recent selection on yeast nitrogen use. *Molecular Biology and Evolution* **32,** 153–161 (2015).

28. Jauniaux, J. C. & Grenson, M. GAP1, the general amino acid permease gene of Saccharomyces cerevisiae. Nucleotide sequence, protein similarity with the other bakers yeast amino acid permeases, and nitrogen catabolite repression. *Eur. J. Biochem.* **190,** 39–44 (1990).
29. Regenberg, B., Düring-Olsen, L., Kielland-Brandt, M. C. & Holmberg, S. Substrate specificity and gene expression of the amino-acid permeases in Saccharomyces cerevisiae. *Curr. Genet.* **36,** 317–328 (1999).
30. Grauslund, M., Didion, T., Kielland-Brandt, M. C. & Andersen, H. A. BAP2, a gene encoding a permease for branched-chain amino acids in Saccharomyces cerevisiae. *Biochim. Biophys. Acta* **1269,** 275–280 (1995).
31. Kispal, G., Steiner, H., Court, D. A., Rolinski, B. & Lill, R. Mitochondrial and cytosolic branched-chain amino acid transaminases from yeast, homologs of the myc oncogene-regulated Eca39 protein. *J. Biol. Chem.* **271,** 24458–24464 (1996).
32. Buckholz, R. G. & Cooper, T. G. The allantoinase (DAL1) gene of Saccharomyces cerevisiae. *Yeast* **7,** 913–923 (1991).
33. Yoo, H. S. & Cooper, T. G. Sequences of two adjacent genes, one (DAL2) encoding allantoicase and another (DCG1) sensitive to nitrogen-catabolite repression in Saccharomyces cerevisiae. *Gene* **104,** 55–62 (1991).
34. Yoo, H. S. & Cooper, T. G. The ureidoglycollate hydrolase (DAL3) gene in Saccharomyces cerevisiae. *Yeast* **7,** 693–698 (1991).
35. Cooper, T. G., Lam, C. & Turoscy, V. Structural analysis of the dur loci in S. cerevisiae: two domains of a single multifunctional gene. *Genetics* **94,** 555–580 (1980).
36. Magasanik, B. & Kaiser, C. A. Nitrogen regulation in Saccharomyces cerevisiae. *Gene* **290,** 1–18 (2002).
37. Naseeb, S. & Delneri, D. Impact of chromosomal inversions on the yeast DAL cluster. *PLoS ONE* **7,** e42022 (2012).
38. Young, A. I. & Durbin, R. Estimation of epistatic variance components and heritability in founder populations and crosses. *Genetics* **198**, 1405–1416 (2014).
39. Yang, J. et al. Common SNPs explain a large proportion of the heritability for human height. *Nat. Genet.* **42**, 565–569 (2010).
40. Yang, J., Lee, S. H., Goddard, M. E. & Visscher, P. M. GCTA: a tool for genome-wide complex trait analysis. *Am. J. Hum. Genet.* **88**, 76–82 (2011).
